# Passing Events and Topics

**Published:** 1921-03-05

**Authors:** 


### Passing Events and Topics.

### A Successful Saturday Fund.

Croydon Hospital Saturday Fund, the operations of which are continuous throughout the year, has handed to the hospital authorities the record sum of £2,650, the net result of collections and donations during 1920.

### London's Ambulance Service.

Deptford Borough Council has complained to the Joint Committee of the Metropolitan Borough Councils with regard to alleged deficiencies in connection with the removal of non-infectious cases, suggesting that this could be met by an extension of the L.C.C. ambulance service. The Joint Committee has made inquiries, and finds that there is no difficulty in obtaining ambulances from the Metropolitan Asylums Board, whose arrangements, however, do not seem to be widely known. The Board, it is revealed, supplies ambulances for the conveyance of medical, surgical, and mental cases. The charge is 10s. within the Metropolitan area, which includes when necessary the services of a male attendant, and in addition 1s. for each mile outside the area, and 2s. 6d. an hour for "waits." Fare and a-half is charged for two patients in an ambulance. Nurses to accompany non-infectious cases cannot be supplied, but a friend may accompany a patient. Hence in these circumstances the Joint Committee comes to the conclusion that the setting-up of a further ambulance service for non-infectious patients does not appear to be required.

### Children Who Do Not Suffer.

Do children suffer during periods of distress? It is difficult to realise that the answer should be in the negative, but this reply is indeed suggested by a report of the Southampton County Medical Officer of Health. He states that on account of the report of considerable unemployment existing in Basingstoke, special attention has been given to the medical inspection of the children of persons said to be unemployed. All the schools were being visited, and the children of unemployed persons specially examined. The weights of the children were compared with those previously recorded on the cards, altogether 138 children being examined. For the purpose of comparison a number of children whose parents were not out of employment were similarly weighed and inspected. According to a report of the Southampton County M.O.H., among the 138 children examined there were only sixteen who showed under-development or arrested development, and on careful examination and investigation there seemed to be no reason to associate this condition with under-feeding at the present time. No marked differences were observed between the weights of the children of the unemployed and other children of the same age.

